# Malaria en poblaciones con ocupación minera, Colombia, 2012-2018

**DOI:** 10.7705/biomedica.5899

**Published:** 2021-05-31

**Authors:** Daniela Salas, Dora Yurany Sánchez, Germán Achury, Fabio Escobar-Díaz

**Affiliations:** 1 Maestría en Salud Pública y Desarrollo Social, Fundación Universitaria del Área Andina, Bogotá, D.C., Colombia Fundación Universitaria del Área Andina Fundación Universitaria del Área Andina BogotáD.C Colombia

**Keywords:** malaria, enfermedades transmitidas por vectores, minería, vigilancia en salud pública, Colombia, Malaria, vector borne diseases, mining, public health surveillance, Colombia

## Abstract

**Introducción.:**

La malaria representa uno de los mayores desafíos de salud pública, principalmente en los países pobres. Ciertas características sociales de Colombia, como la migración, el trabajo informal y la escasez económica, favorecen la minería ilegal. El análisis de la situación de la malaria en estas áreas permite establecer una guía para la prevención, el control y el tratamiento de la enfermedad en los programas de salud pública existentes.

**Objetivo.:**

Describir la situación de la malaria en las poblaciones mineras colombianas entre el 2012 y el 2018.

**Materiales y métodos.:**

Se hizo un estudio descriptivo y retrospectivo que incluyó la creación de gráficos y mapas. Para el análisis estadístico se utilizaron la correlación de Pearson y el índice de Moran.

**Resultados.:**

Entre el 2012 y el 2018, se notificaron 44.032 casos de malaria en la población minera, 43.900 de malaria no complicada y 132 de malaria complicada, así como tres muertes, dos por *Plasmodium vivax* y una por infección mixta. Hubo una disminución del 44,7 % de los casos en el período evaluado. La tasa de riesgo en el 2012 fue de 2,5 casos por 1.000 habitantes; el 87,3 % de los casos se presentó en hombres y el 37,9 % en personas entre los 20 y los 29 años, en tanto que el 46,7 % de la población estudiada estaba conformada por afrocolombianos. Se encontró una posible correlación lineal positiva moderada entre mayor la actividad minera, mayor el número de casos de malaria en mineros. El índice de Moran global evidenció una agrupación espacial significativa de los casos de malaria en zonas con industria minera en los municipios del Pacífico colombiano.

**Conclusiones.:**

La disminución en la notificación de casos durante el período evaluado podría atribuirse a un subregistro del Sistema de Vigilancia en Salud Pública (Sivigila), ya que la mayoría de los mineros no tienen trabajos formales, lo que dificulta su acceso a los servicios de salud. Se recomienda un estudio de cohorte en áreas endémicas para establecer una relación directa entre la explotación minera y la presencia de casos de malaria.

## Abstract (en)

**Introduction:**

Malaria represents one of the biggest public health challenges, mainly in poor countries. Colombia has social characteristics such as migration, informal work, and economic shortages that favor illegal mining activities. The study of the malaria situation in these areas would allow establishing the bases for its prevention, control, and treatment in the existing public health programs.

**Objective:**

To describe the malaria situation in Colombian mining populations between 2012 and 2018.

**Materials and methods.:**

We conducted a retrospective descriptive study with graphs and maps. For the statistical analysis, we used Pearson's correlation and Moran's index.

**Results.:**

From 2012 to 2018, 44,032 cases of malaria were reported in the mining population, 43,900 of uncomplicated malaria and 132 of complicated malaria, and three deaths, two due to *Plasmodium vivax* and one due to mixed infection. During this period, there was a decrease of 44.7% in cases. The risk rate in 2012 was 2.5 cases x 1000 inhabitants; 87.3% of cases were in men, and 37.9% corresponded to the 20 to 29-year-old age group while 46.7% were AfroColombians. We found a possible moderate positive linear correlation: The greater the mining activity, the greater the number of malaria cases. The global Moran index indicated a significant spatial grouping of cases in mining activities in Colombian Pacific municipalities.

**Conclusions.:**

The case notification decrease during this period could be attributed to an underreporting of the public health surveillance system (Sivigila) system, as most miners do not have formal jobs, which prevents them from accessing health services. A cohort study is recommended in endemic areas to establish a direct relationship between mining exploitation and the occurrence of malaria cases.

La malaria o paludismo es una enfermedad infecciosa generada por una variedad de parásitos protozoarios pertenecientes al género *Plasmodium* y transmitida por la hembra del mosquito *Anopheles.* Se han identificado cinco especies distintas de plasmodios que infectan a los seres humanos: *P. falciparum, P. vivax, P. malariae, P. ovale* y *P. knowlesi*^1^. La Organización Mundial de la Salud (OMS) diseñó la Estrategia Técnica Mundial contra la Malaria, 2016-2030, un ambicioso plan que aspira a lograr el control y la eliminación de la enfermedad, y cuyo objetivo global es reducir en más del 40 % la incidencia y la tasa de mortalidad para el 2030 ^2^.

En Latinoamérica, la malaria constituye uno de los grandes desafíos de salud pública, pues se la considera endémica en la mayor parte de los países y afecta la salud de aproximadamente 102 millones de personas que se creen en riesgo de contraer la enfermedad ^3^, principalmente en países pobres y marginados. Se afirma que hoy el impacto de la malaria va más allá del número de fallecimientos, pues se ha convertido en un factor limitante para el progreso de las sociedades asociado con situaciones de pobreza y retraso del desarrollo social, escolar y laboral, con la consiguiente imposibilidad de lograr un adecuado crecimiento económico en los diferentes territorios donde es endémica.

Durante las últimas dos décadas, cerca del 40 % del territorio colombiano ha sido objeto de la explotación de minerales e hidrocarburos ^4^, actividad que se ha consolidado como uno de los sectores estratégicos para la economía nacional, ya que es responsable de financiar cerca del 85,4 % del gasto público y contribuye con el 7 % al producto interno bruto (PIB) ^5^. Sin embargo, la ubicación de las minas en zonas endémicas para malaria hace que los costos del tratamiento, la incapacidad laboral y los desenlaces fatales contribuyan a la disminución del ingreso familiar y afecten los esfuerzos del Estado para su control, lo que termina por perjudicar la economía nacional ^6^.

Este panorama ha estado acompañado por la rápida expansión de la minería ilegal, lo que expresa un problema de orden social e informalidad que tiene un importante impacto económico y de salud pública, puesto que solo cerca del 20 % de las minas registradas en Colombia cuentan con título minero ^7^. Además, la minería ilegal se ha vinculado al patrocinio de actividades igualmente ilícitas, como la financiación del terrorismo y de bandas dedicadas al narcotráfico, entre otros ^5^.

El vínculo entre la malaria y la población dedicada a las actividades mineras, es uno de los problemas de salud en el que confluyen factores sociales de gran impacto, cuya eliminación requiere de medidas de gran alcance destinadas, no solo al control de la enfermedad, sino también a comprender particularidades de la población, como los fenómenos de migración que favorecen la circulación de personas infectadas con malaria entre los sitios donde se ejerce la minería, además de características ambientales, como la altitud, el clima, o la intensidad de la lluvia, que favorecen la adaptación de los mosquitos anófeles, y características clínicas, como las infecciones asintomáticas o la automedicación que da paso a la resistencia a los medicamentos ^8^.

La malaria afecta de forma importante a las personas que ejercen la minería de forma ilegal, factor que pone de manifiesto el carácter hiperendémico de las áreas donde se lleva a cabo esta actividad. La automedicación sistemática por parte de los pacientes que utilizan artemisinina y otros fármacos similares sin seguir el ciclo completo de tratamiento, es un riesgo grave que aumenta la resistencia a los medicamentos empleados contra las infecciones por *P. vivax* y P *falciparum*^9^.

Es indispensable un nuevo enfoque para comprender integralmente los cambios en el panorama epidemiológico de la malaria en Colombia, lo que incluye abordar la población dedicada a los diferentes tipos de minería en el país notificada en el Sivigila entre el 2012 y el 2018. Asimismo, deben emprenderse análisis cuyos resultados contribuyan a la formulación o reorientación de las políticas públicas de prevención, control y tratamiento de la malaria en esta población, así como a implementar las recomendaciones de estudios previos en torno al robustecimiento de los programas en salud pública existentes, haciéndolos más funcionales y propiciando una mayor sensibilización de las instituciones estatales y de la población que practica la minería en el territorio colombiano ^8^^,^^10^.

En este contexto, el objetivo del estudio fue describir la situación de la malaria en la población minera en Colombia entre el 2012 y el 2018. Se determinaron las características sociales y demográficas de la población minera afectada por la malaria, y se estimó la relación existente entre la explotación minera y la aparición de casos.

## Materiales y métodos

Se hizo un estudio descriptivo y retrospectivo con un universo de 407.795 casos de malaria registrados en las bases de datos del Sivigila en la plataforma del Instituto Nacional de Salud ^11^ entre la semana epidemiológica 01 de 2012 y la semana epidemiológica 52 de 2018.

Los datos corresponden a casos confirmados de malaria notificados por los servicios de salud del país, de los cuales se seleccionaron solo 44.032 (10,79 %) para el análisis después de excluir aquellos que no correspondían a la población minera, así como los registros duplicados y que no tuvieran la información completa. Los datos de producción de oro del país se tomaron del Sistema de Información Minero Energético Colombiano, publicados como datos abiertos en su sitio Web para los años 2012 a 2018; la información sobre minería ilegal se tomó de los mapas publicados por la Asociación Colombiana de Minería ^12^.

Se diseñó y validó una base de datos en Microsoft Excel para almacenar la información recopilada. Las variables epidemiológicas y mineras definidas para el estudio fueron: número total de casos de malaria de 214 municipios, especies de parásitos, población en riesgo, y producción anual de oro por municipios y distritos mineros (163 municipios) durante el período de estudio.

El índice parasitario anual se calculó utilizando el número total de casos de malaria entre la población con ocupación minera en cada municipio según el Sivigila y la población total en riesgo por municipio a nivel nacional para cada año del período de estudio según el Ministerio de Salud y Protección Social, la cual se calculó por cada 1.000 habitantes.

Se usó el programa IBM SPSS Statistics Base 22.0™ para hacer el análisis estadístico y de correlación de Pearson. El programa Microsoft Excel™ se empleó para crear gráficos, en tanto que los mapas y el análisis de Moran se hicieron con ArcGis 10.8™.

Los datos de distribución de la población por año y municipios, así como los *shapefiles* de Colombia, se obtuvieron del Departamento Administrativo Nacional de Estadística (DANE) ^13^ y se utilizaron como base para el análisis espacial encaminado a determinar la distribución de los casos de malaria y de las minas de oro en Colombia.

La significación estadística se estableció con probabilidades menores del 5 % (p<0,05). Para el análisis de los datos, se recurrió a una correlación lineal de Pearson, con el fin de establecer la relación entre la producción minera de oro y los casos notificados de malaria entre el 2012 y el 2018 en los 214 municipios de explotación minera de oro que presentaron, por lo menos, un caso de malaria.

Las bases de datos se depuraron siguiendo las recomendaciones del sistema de vigilancia (Sivigila) con base en las reglas de validación, código variable, fecha de notificación, unidades generadoras de datos primarios y unidades notificadoras, lo que permitió la evaluación de los casos duplicados. Se estableció un plan de análisis de las variables para calcular las frecuencias absolutas y relativas.

En cuanto al análisis estadístico espacial, se aplicó el índice global de Moran para verificar el patrón espacial de los casos de malaria notificados en la población minera, y se hizo la autocorrelación espacial mediante el análisis de conglomerados y valores atípicos para determinar puntos calientes, puntos fríos y valores atípicos espaciales estadísticamente significativos (p<0,05) con el uso del programa ArcGIS 10.8.

Conforme a lo dispuesto en la Resolución 8430 de 1993 del Ministerio de Salud y Protección Social de Colombia, que define las normas científicas, técnicas y administrativas para la investigación en salud, este estudio se clasificó como una investigación sin riesgo debido a su carácter retrospectivo que no contempla intervenciones o modificaciones intencionadas en las variables biológicas, fisiológicas, psicológicas o sociales.

## Resultados

Entre el 2012 y el 2018, se notificaron a nivel nacional 407.795 casos de malaria, de los cuales el 46,6 % se debió a infecciones por *P. falciparum,* el 51,4 % por *P. vivaxy* el 2 % por infección mixta, con 5.443 casos de malaria complicada, 402.497 casos de malaria no complicada y 137 muertes.

**Figura 1:**
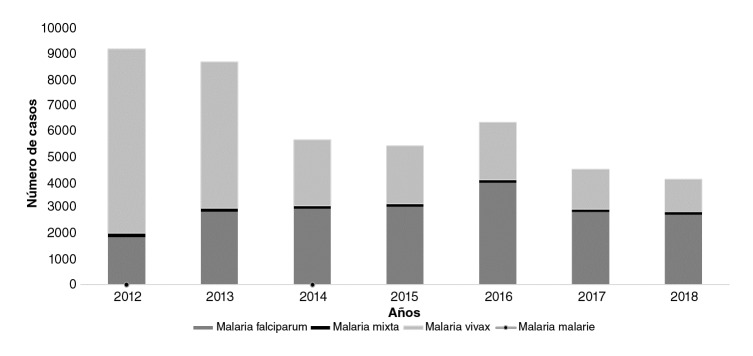
Distribución de casos de malaria en población minera, Colombia, 2012-2018

En el periodo se notificaron 44.032 casos de malaria en la población minera, de los cuales 43.900 fueron de malaria no complicada, 132 de malaria complicada y tres muertes, dos por *P. vivaxy* una por infección mixta. Predominó la infección por *P. vivax,* con 51,87 % (22.841 casos), seguida por *P. falciparum,* con 46,2 % (20.344 casos). Los casos de malaria en la población minera disminuyeron progresivamente en un 44,7 % entre el 2012 y el 2018 (figura 1).

Fuente: Sivigila, Instituto Nacional de Salud, Colombia

Los departamentos de Chocó, Antioquia, Bolívar y Nariño registraron el 90,5 % de los casos de malaria no complicada en población minera. El riesgo epidemiológico de malaria para los 214 municipios con este tipo de casos en el 2012, fue de 2,5 casos por cada 1.000 habitantes en riesgo (población del área rural) y, en el 2018, fue de un caso por cada 1.000 habitantes en riesgo. Los casos se concentraron en la población masculina, con 38.479 casos (87,3 %). La distribución por edad mostró que el grupo de 20 a 24 años fue el más afectado con 8.506 casos (19,3 %); seguido del grupo de 25 a 29 años con 8.231 casos (18,6 %). En cuanto a la pertenencia étnica, 20.574 casos (el 46,7 %) se notificaron como población afrocolombiana; en la población indígena, por su parte, la notificación fue solo de 1.322 casos (el 0,03 %) y 22.046 (51,1 %) casos no correspondían a ninguna población étnica específica.

**Figura 2:**
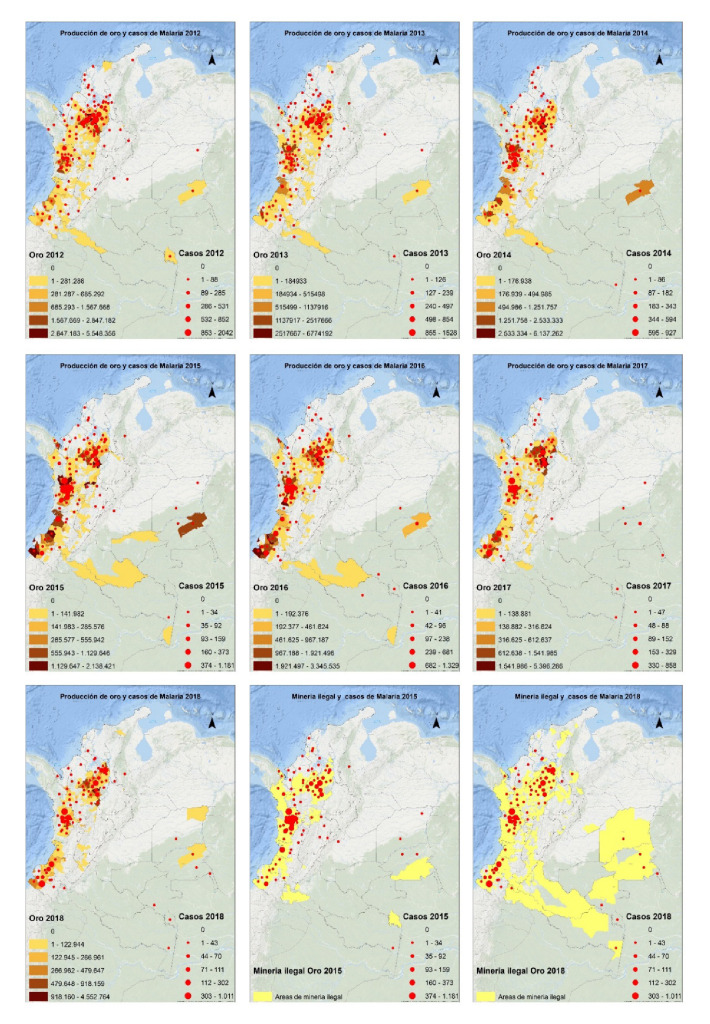
Distribución de casos de malaria, producción de gramos de oro por municipio y municipios con reporte de minería ilegal, Colombia, 2012-2018

Según lo reportado por la Agencia Nacional de Minería, los municipios de El Bagre, Segovia y Remedios en Antioquia, y Quibdó en Chocó, son los de mayor extracción legal de oro del país. Sin embargo, en las mismas zonas donde se realiza esta actividad de forma legal, con el pasar de los años se ha percibido un aumento de la minería ilegal, especialmente en el Pacífico y la Amazonia. En estos mismos municipios se registraron casos de malaria, siendo los de mayor número de casos acumulados Quibdó (Chocó) con 5.958 casos, El Bagre (Antioquia) con 4.979 casos y Barbacoas (Nariño) con 2.937 casos (figura 2).

Fuente: Sivigila, Instituto Nacional de Salud y SIMEC, Unidad de Planeación Minero-Energética, Colombia

**Figura 3:**
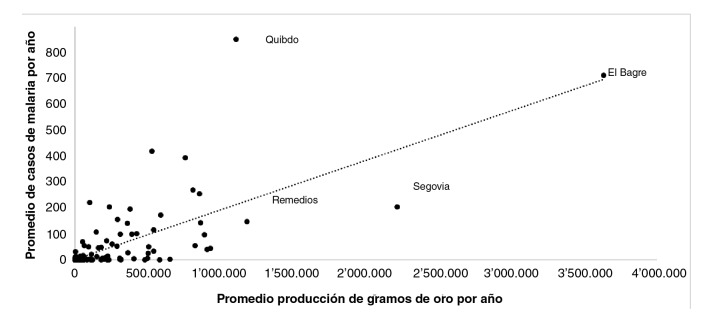
Dispersión de producción minera de oro y casos de malaria en población minera, Colombia, 2012-2018

En el análisis de correlación lineal de Pearson, se encontró una posible correlación lineal positiva moderada, con un coeficiente R^2^ de 0,5, lo que nos indicaría que se espera que, a mayor producción minera de oro, mayor sea el número de casos de malaria en la población minera; los datos presentaron una alta dispersión, siendo los municipios de El Bagre, Segovia, Remedios y Quibdó los de mayor producción de oro durante los años del estudio, por encima del millón de gramos por año (figura 3).

Fuente: Slviglla, Instituto Nacional de Salud y SIMEC, Unidad de Planeaclón Minero-Energética, Colombia

En cuanto al análisis estadístico espacial, se aplicó el índice global de Moran para verificar el patrón espacial de los casos de malaria que mide la autocorrelación espacial basada en las ubicaciones y los valores de las entidades simultáneamente, y evalúa si el patrón expresado esta agrupado, disperso o es aleatorio. El índice global de Moran indicó una agrupación espacial significativa de los casos de malaria en población minera; la autocorrelación espacial evidenció un grupo bajo-bajo en la región del Caribe y centro del país, y es importante aclarar que en estas zonas se presentó el 0,05 % de los casos de malaria en población minera; además, otro grupo alto-alto y otro alto-bajo, con el 81,9 % del total de los casos analizados (36.065/44.032 casos) ubicado en el 19,2 % de los municipios con casos de malaria (42/214 municipios).

**Figura 4:**
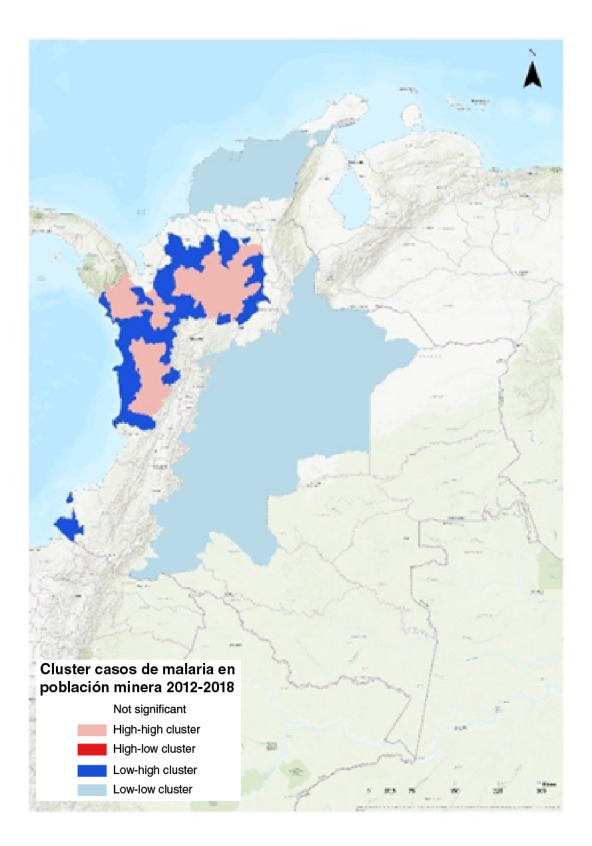
Distribución del análisis de conglomerados y valores atípicos (Anselin Local Moran I) para casos de malaria en población minera en Colombia, 2012-2018

Dada la puntuación Z de 11,69, este resultado evidenció que existe una probabilidad menor del 1 % de que el patrón de agrupación pueda ser producto de una verosimilitud aleatoria, por lo tanto, los datos estadísticos permiten afirmar que los casos de malaria siguieron un patrón de distribución agregado (figura 4).

Fuente: Sivlgila, Instituto Nacional de Salud, Colombia

## Discusión

El objetivo del estudio era describir la situación de la malaria en la población con ocupación minera en Colombia entre el 2012 y el 2018. Según los resultados obtenidos, se pudo establecer una posible correlación entre la explotación minera de oro y los casos de malaria, lo que permite inferir los datos a los individuos que habitan en los municipios con casos de malaria y con explotación de oro. En algunos estudios en países donde se práctica la minería, como Brasil, se ha encontrado una asociación entre la extracción de oro y el incremento en la incidencia parasitaria anual ^14^.

En el mapeo de las zonas mineras del país, se estableció una mayor frecuencia de población con malaria en el Pacífico colombiano, con una posible correlación lineal positiva moderada en algunos municipios de esta región. En un estudio del Observatorio Nacional de Salud en el Chocó, el departamento con mayor producción aurífera del país, se encontró una importante asociación entre los casos de malaria y la producción de oro ^15^. En otro estudio similar, se ubicaron las minas de oro ilegales mediante datos satelitales del país y estos coincidieron con informes oficiales sobre casos de malaria, lo que sugiere que cuando un área contiene minas de oro ilegales en una hectárea, aumenta el índice parasitario anual ^16^.

En el presente estudio, se estableció que el 87 % de los casos se dio en hombres, y el 38 % eran personas en la etapa productiva, con edades entre los 20 y los 29 años; esta característica es frecuente en las actividades mineras que requieren de gran esfuerzo físico. Algunos estudios sugieren que los hombres tienen un riesgo mayor de presentar malaria, lo que probablemente se relaciona con factores que afectan la exposición al mosquito, como la extracción minera de oro a cielo abierto ^15^.

Además, el 12,8 % de la población es mayor de 50 años, un grupo de edad que se encuentra al final de su etapa productiva; también, se estableció la presencia de mujeres, niños y adolescentes que registraron la minería como su principal ocupación y, a veces, como su única fuente de sustento, debido a la baja escolaridad y la ausencia de afiliación a la seguridad social, perpetuando esta forma de vida para las siguientes generaciones ^17^. Además, la falta de medidas eficaces de control, su desconocimiento cuando las hay, la proximidad de su alojamiento y la migración propia de estos trabajadores, contribuyen a la propagación de enfermedades como la malaria ^8^.

Según el censo nacional minero de 2010-2011, de 4.133 minas de oro censadas, solo 549 (13,3 %) poseían título minero, por lo que esta actividad en el país la desarrollan mayoritariamente mineros artesanales sin formalización laboral ^17^, factor que dificulta el acceso a los servicios de salud porque no están afiliados al sistema general de seguridad social en salud y tiene implicaciones a la hora de acceder al tratamiento oportuno; además, se ha establecido que las personas con mayor número de necesidades básicas insatisfechas tienen mayor riesgo de presentar malaria ^15^.

El descenso en la notificación de los casos al Sivigila en el periodo evaluado podría atribuirse a un subregistro y a la ausencia del reporte porque la vigilancia epidemiológica es pasiva ^18^. En un estudio de captura y recaptura, se evidenció un subregistro del 80 % en la notificación de este evento ^19^, lo que subraya la necesidad de mejorar los procesos de capacitación del personal encargado de la recopilación de datos, mediante la implementación de cursos regulares, el fortalecimiento de los procedimientos de sensibilización y la presentación oportuna de informes ^20^.

El presente estudio entrega un panorama de la situación actual de la malaria en la población minera y se puede inferir que los casos siguen un patrón de distribución agregado. Se recomienda hacer un estudio de cohortes en las zonas afectadas que permita establecer una relación directa entre la explotación minera y la presentación de casos de malaria. Los resultados que aquí se reportan podrían emplearse como punto de partida para la construcción de una política pública coherente y estructurada, que involucre de forma integral aspectos ambientales, sociales, económicos, laborales, de salud pública y ocupacional con énfasis en poblaciones vulnerables ante la evidente desarticulación institucional que se presenta en torno al fenómeno de la minería ilegal en el país ^10^. Se debe tener en cuenta el contexto actual de las personas ocupadas en la minería con énfasis en la sensibilización frente al riesgo, y el fortalecimiento de las actividades de promoción, prevención, detección precoz y oportunidad del tratamiento en esta población a partir de su contexto particular.
